# Exploring the Amino-Acid Composition, Secondary Structure, and Physicochemical and Functional Properties of Chickpea Protein Isolates

**DOI:** 10.1021/acsomega.2c06912

**Published:** 2022-12-20

**Authors:** Sumeyra Onder, Asli Can Karaca, Beraat Ozcelik, Abdulhakeem S. Alamri, Salam A. Ibrahim, Charis M. Galanakis

**Affiliations:** † Department of Food Engineering, Faculty of Chemical and Metallurgical Engineering, Istanbul Technical University, 34469 Istanbul, Turkey; ‡ Department of Clinical Laboratory Sciences, College of Applied Medical Sciences, Taif University, Taif 26571, Saudi Arabia; § Centre of Biomedical Sciences Research (CBSR), Deanship of Scientific Research, Taif University, Taif 26571, Saudi Arabia; ∥ Food and Nutritional Sciences Program, North Carolina A&T State University, Greensboro, North Carolina 27411, United States; ⊥ Department of Research & Innovation, Galanakis Laboratories, Skalidi 34, 73131 Chania, Greece; # Department of Biology, College of Science, Taif University, Taif 26571, Saudi Arabia; ∇ Food Waste Recovery Group, ISEKI Food Association, Vienna 1190, Austria

## Abstract

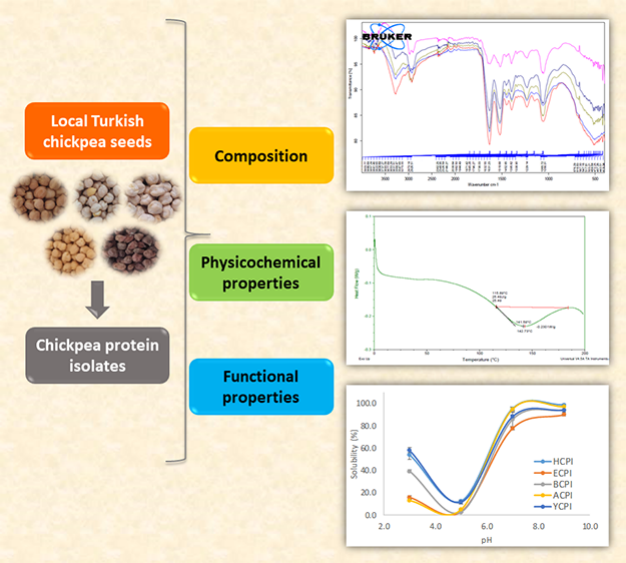

This study examined the amino-acid profile, secondary structure, and physicochemical and functional properties of proteins isolated from Anatolian chickpea landraces. Secondary objective of the study was to determine whether a relationship exists between the amino-acid composition and physicochemical and functional properties. Aspartic acid and glutamic acid were the dominant amino acids, while the isolates were deficient in methionine. Secondary structures were determined by Fourier transform infrared spectroscopy, where the β-sheet was shown to be dominant. The denaturation temperature of the isolates was between 87 and 145 °C, and the highest net surface charge (≃28.6 mV) and solubility (∼95.0%) were observed at pH 9.0–10.0. The isolates’ water-holding capacity varied between 2.1 and 2.7 g water/g protein, whereas their oil-holding capacity ranged between 3.4 and 4.4 g oil/g protein. Emulsion capacity, emulsifying activity, and the stability indices of isolates were found to be between 401.2 and 469.1 g oil/g protein, 14.5 and 25.7 m^2^/g, and 45.7 and 146.9 min, respectively. Isolates of Hisar and Erzincan chickpeas exhibited good emulsifying properties. The Yasa isolate had a relatively high hydrophobic amino-acid content and delivered the best gelation performance. Overall, significant differences in the characteristics of proteins were observed among the different chickpea landraces studied.

## 1 Introduction

Following the catastrophic effects of the global COVID-19 pandemic, consumers have become more attentive to healthier diet choices, while food manufacturers are seeking more sustainable food ingredient sources such as plant-based proteins.^1−3^ Most of the world’s protein is supplied from cereals, legumes, and oilseeds. As these plant protein sources are less costly than others, their use in the food industry is increasing. Moreover, selecting appropriate protein extraction methods is essential because this affects the composition and nutritional and functional properties of the final product.^4−6^ The functional properties of proteins such as solubility, viscosity, water, fat holding, foaming, emulsion, and gelling properties affect the textural and organoleptic properties of food products.^4,7^ The cultivation of chickpeas (*Cicer arietinum* L.) in Turkey dates back to 7000 years ago.^8^ Chickpea protein isolates can be recovered using several techniques including isoelectric precipitation, ultrafiltration, and ultrasounds to separate macro- and micromolecules.^9,10^ Other techniques such as salt extraction and wet-milling techniques have also been used to investigate the structural and functional properties of these isolates.^4,11−20^ Boye and co-workers^4^ investigated the physicochemical and functional properties of proteins extracted from several pulses, including Desi and Kabuli chickpeas. Aydemir & Yemenicioglu^16^ characterized the functional properties of Turkish Kabuli-type chickpea proteins and compared them with soy and animal proteins. In a recent study, Tontul et al.^19^ investigated the effect of the drying method on surface hydrophobicity and functional properties of chickpea protein isolates. Moreover, Wang et al.^21^ and Xu et al.^22^ applied ultrasound treatment for the improvement of emulsifying and gelling properties of chickpea protein isolates. In another recent study, Mesfin and co-workers^23^ investigated the effects of germination, roasting, and variety on the physicochemical, functional, and antioxidant properties of isolates obtained from Arerti (Kabuli type) and Natoli (Desi type) chickpea varieties from Ethiopia, whereas Zhang and co-workers^24^ investigated the functional and nutritional properties of isolates from Kabuli type of chickpea from China.

Providing information on protein composition and functionality has an essential role in the evaluation of the potential of local landraces to be used as plant-based protein sources. It has been indicated that protein isolates obtained from different cultivars can show significant differences in composition and characteristics.^4,13,15^ There are several reports on the characterization of structural, physicochemical, and functional properties of proteins obtained from various chickpea cultivars. Chickpea is an important agricultural commodity for Turkey. However, to the best of our knowledge, proteins extracted from the Hisar, Erzincan, black chickpea, Azkan, and Yasa, which are local chickpea landraces of Anatolia, have not been characterized. The current study thus aimed to determine the amino-acid profile, secondary structure, net surface charge, and thermal and functional properties of isoelectrically precipitated proteins from local Turkish chickpea landraces. The secondary objective of the study was to evaluate if a relationship exists between the amino-acid composition and the physicochemical or functional properties of the isolates. The novelty of the present study lies in revealing the relationship between the amino-acid composition and physicochemical and functional properties of proteins isolated from different chickpea cultivars, which can provide valuable information for valorization of specific agricultural commodities.

## 2 Materials and Methods

### 2.1 Materials

Seeds from Azkan, Yasa, and Hisar chickpeas harvested in 2017 were obtained from the Directorate of Provincial Agriculture and Forestry (Eskisehir, Turkey), and black chickpea and Erzincan chickpea seeds harvested in 2017 were obtained from local markets. Sunflower seed oil was supplied from a local market in Istanbul, Turkey. All chemicals used in this study were of reagent grade (Sigma-Aldrich, St. Louis).

### 2.2 Preparation of Chickpea Protein Isolates

Chickpea protein isolates were obtained from defatted chickpea flour according to the alkaline extraction (pH 9.0) and isoelectric precipitation (pH 4.6) method of Papalamprou et al.^25^ and freeze-dried at −40 °C for 24 h (α 1-2 LD plus, Martin Christ Gefriertrocknungsanlagen GmbH, Osterode am Harz, Germany). The obtained isolate powders were stored in tightly packed glass jars at 4 °C until further use.

### 2.3 Proximate Composition

The Association of Official Analytical Chemists method was used to determine the proximate composition of chickpea flours and protein isolates.^26^ A nitrogen conversion factor of 6.25 was used to calculate the protein content.

### 2.4 Amino-Acid Composition

Shimadzu high-performance liquid chromatography (HPLC) system (Shimadzu Scientific Instruments Inc., Columbia, Maryland 21046) with an auto-sampler (SIL 20ACHT) was used to determine the amino-acid composition of chickpea protein isolates according to the method described in Gundogan & Can Karaca.^27^

### 2.5 Physicochemical Properties

Net surface charge (ζ-potential) measurement of chickpea protein isolates as a function of pH was performed according to the method of Can Karaca et al.^14^ In brief, a solution containing 0.5 g/L protein was prepared, and pH was adjusted using 1.0 mol/L NaOH or 1.0 mol/L HCl. ζ-potential values were determined using a Zetasizer device (Nano-ZS, Malvern Instruments, Malvern, U.K.) based on electrophoretic mobility solutions.

Thermal properties were determined by the method of Ghribi et al.^17^ using a differential scanning calorimetry (DSC) instrument (Model Q10, TA Instruments Inc., New Castle). Differential scanning calorimetry (DSC) curves were recorded with a scanning rate of 5 °C/min during heating from 0 to 200 °C. Onset temperature (*T*_o_), denaturation temperature (*T*_d_), and denaturation enthalpy (Δ*H*) were calculated using Universal Analysis 2000 Version 4.5A (TA Instruments Inc., New Castle). Fourier Transform Infrared (FTIR) spectroscopy was performed using the method of He et al.^28^ The isolate powder was placed in an FTIR spectrophotometer diffuse reflectance device (FTIR Tensor II, Bruker Optics Inc., Billerica), and the measurements were performed at 400–4000 cm^–1^ wavelength range and 4 cm^–1^ resolution. The secondary structure percentage (α-helices, β-layers, etc.) was determined using the relative integral area of each convenient curve of the amide I region. The spectra’s band assignment was designated based on a previous report.^15^

### 2.6 Functional Properties

The solubility of the isolates was determined at pH (3.0–9.0) by the method of Ghribi et al.,^17^ and water-holding (WHC) and oil-holding capacities (OHC) were measured according to Aydemir & Yemenicioglu.^16^

Emulsion capacity (EC), which indicates the amount of oil emulsified per g of protein, was measured at pH 7.0 according to the method of Can Karaca et al.,^14^ with slight modifications described by Gundogan & Can Karaca.^27^ In addition, emulsion activity (EAI) and stability indices (ESI) were determined at pH 7.0 via a spectrophotometric method.^29^

The foaming capacity (FC) and foaming stability (FS) were determined by the method of Ghribi et al.^17^ using 30 g/L protein dispersions at pH 7.0. FC was recorded as the volume increases due to whipping. FS was determined as the change of foam volume after the foaming process at 10, 30, and 60 min of storage.

The gelation capacity was determined by the method of Aydemir & Yemenicioglu^16^ using protein suspensions with concentrations between 10 and 140 g/L at pH 7.0. Gelation capacity was determined based on the lowest protein concentration that yielded a gel without gravity drop or slips after the tubes were inverted.

### 2.7 Statistical Analyses

All measurements were performed in triplicate. Statistical differences were determined with a one-way analysis of variance (ANOVA) and a Tukey’s multiple comparison test, while statistical significance was accepted at *p* < 0.05. Simultaneous multiple regression analysis was conducted to examine the relationship between the amino-acid composition, physicochemical characteristics, and functional properties. Pearson correlation coefficients (*r*) were also calculated to describe the relationship between the amino-acid composition and the physicochemical and functional properties of isolates. All results were analyzed with IBM SPSS Statistics software (version 27.0, IBM, New York).

## 3 Results and Discussion

### 3.1 Proximate Composition of Chickpea Flours and Protein Isolates

The protein content of the chickpeas varied between 20.0/100 g and 24.8/100 g (Table 1), which was found to be following the range of 16–25/100 g previously reported.^4,14,30^ Differences in the protein content of cultivated chickpeas are indicated to depend on the seed type.^31^ Azkan chickpeas were observed to contain the highest amount of protein (∼24.8/100 g). Chickpea flour moisture content varied between 9.5 and 10.8/100 g, whereas their ash content varied between 2.6 and 3.3/100 g. Hisar chickpeas were observed to contain the highest amount of ash. The crude fat content of chickpea flours ranged from 2.9/100 to 4.6/100 g. The crude fat of chickpea flours was reported to be 1.5–7.5/100 g.^4,12,30^ Azkan chickpeas, which contained the highest protein, were observed to have the lowest fat level. The total carbohydrate content of the flours ranged between 59.4 and 62.9/100 g. The carbohydrate content of Erzincan, Black chickpea, and Yasa chickpeas was found to be higher compared to Hisar and Azkan chickpeas. The nutritional composition of chickpeas depends on many different factors, including cultivar, environmental conditions, and maturity stage at harvest.^32^ The protein content of isoelectrically precipitated chickpea protein isolates ranged from 85.9 to 90.2/100 g on a wet basis, and the protein extraction yield was between 53.9 and 61.4/100 g. In a study by Boye et al.,^4^ the protein content of an isoelectrically precipitated chickpea protein isolate was reported as 73.6/100 g, and the extraction yield was 53.7/100 g. The crude fat content of the isolates ranged between 0.7 and 1.2/100 g. In another study by Kaur & Singh,^13^ the oil content of chickpea protein isolates was in the range of 0.5–0.9/100 g. The ash content of protein isolates ranged between 2.2 and 3.3/100 g. Sanchez-Vioque and co-workers^12^ reported the ash content of chickpea protein isolates to be between 0.8 and 1.1/100 g. Total carbohydrate content of the isolates ranged from 0.7 to 6.9/100 g, where Black chickpea isolate had the lowest amount of carbohydrate and Hisar isolate had the highest. Although the presence of carbohydrates is indicated to interfere with the protein extraction process in legumes, no direct correlation between carbohydrate content and protein extraction yield was observed (*p* > 0.05).

**Table 1:** Proximate Composition (g/100 g) of Chickpea Flours and Protein Isolates (as is Basis) and Protein Extraction Yield (%)

sample	protein	moisture	ash	crude fat	carbohydrate^b^	protein extraction yield^c^(%)
chickpea flours						
hisar	21.4 ± 0.2^b^^a^	10.8 ± 0.1^a^	3,3 ± 0.1^a^	4.1 ± 0.3^ab^	60.5 ± 0.5^b^	
erzincan	20.0 ± 0.1^c^	10.1 ± 0.2^bc^	2,6 ± 0.1^d^	4.6 ± 0.2^a^	62.7 ± 0.4^a^	
black chickpea	21.1 ± 0.4^bc^	9.5 ± 0.3^d^	2.6 ± 0.1^cd^	3.9 ± 0.4^ab^	62.9 ± 0.7^a^	
azkan	24.8 ± 0.8^a^	9.9 ± 0.3^cd^	3.1 ± 0.1^b^	2.9 ± 0.1^c^	59.4 ± 0.9^b^	
yasa	20.4 ± 0.1^bc^	10.6 ± 0.1^ab^	2.8 ± 0.1^c^	3.4 ± 0.2^bc^	62.7 ± 0.3^a^	
chickpea protein isolates						
hisar	85.9 ± 0.8^c^	4.3 ± 0.3^b^	2.2 ± 0.1^c^	0.7 ± 0.1^b^	6.9 ± 0.9^a^	56.9 ± 1.0^b^
erzincan	88.8 ± 0.8^ab^	4.1 ± 0.5^b^	2.7 ± 0.1^b^	0.9 ± 0.1^b^	3.5 ± 0.2^c^	61.4 ± 1.1^a^
black chickpea	90.2 ± 0.3^a^	5.8 ± 0.3^a^	2.5 ± 0.1^bc^	0.7 ± 0.1^b^	0.7 ± 0.4^d^	56.0 ± 0.1^bc^
azkan	86.8 ± 0.9^bc^	3.8 ± 0.4^b^	3.3 ± 0.1^a^	0.8 ± 0.1^b^	5.3 ± 0.7^b^	57.9 ± 1.2^b^
yasa	86.7 ± 0.4^c^	4.4 ± 0.2^b^	2.3 ± 0.3^c^	1.2 ± 0.1^a^	5.4 ± 0.6^ab^	53.9 ± 0.5^c^

a Data represent the mean ± SD (*n* = 3); mean values with different letters within the same column are significantly different.

b Calculated by percentage differential from 100%.

c Yield of protein isolate was determined according to the method of Makeri et al.^51^

### 3.2 Amino-Acid Composition

The amino-acid profile of isolates (Table 2) indicated that glutamic and aspartic acids were the dominant amino acids, followed by arginine, leucine, lysine, and phenylalanine. This was similar to what was noted in previous reports for other landraces.^33−35^ However, significant differences were observed in the hydrophobic amino-acid groups. The Yasa isolate was observed to contain relatively higher amounts of hydrophobic amino acids (32%) than the other isolates (∼28%). Leucine, lysine, and phenylalanine were the primary essential amino acids. However, according to the FAO/WHO/UNU requirements, the isolates were deficient in methionine. Since chickpea protein isolates are rich in lysine and low in methionine, complementing the isolates with protein sources with lower lysine/ higher methionine levels has been suggested for an improved amino-acid profile.^33,35^

**Table 2:** Amino-Acid Composition (g/16 g N) of Chickpea Protein Isolates

amino acid	Hisar isolate	Erzincan isolate	Black chickpea isolate	Azkan isolate	Yasa isolate	FAO/WHO requirements^a^
nonessential amino acids						
Asp	12.3 ± 0.4^ab^^b^	12.6 ± 0.4^ab^	11.4 ± 0.1^b^	13.0 ± 0.8^a^	11.7 ± 0.3^ab^	
Glu	14.2 ± 0.3^b^	16.7 ± 0.7^a^	15.3 ± 0.3^ab^	14.5 ± 0.4^b^	16.6 ± 0.6^a^	
Ser	5.0 ± 0.7^a^	4.8 ± 0.5^a^	4.6 ± 0.3^a^	5.2 ± 0.3^a^	4.9 ± 0.3^a^	
Gly	3.9 ± 0.6^a^	3.7 ± 0.1^a^	3.8 ± 0.2^a^	3.6 ± 0.2^a^	3.5 ± 0.1^a^	
Arg	7.9 ± 0.3^b^	9.3 ± 0.4^a^	8.7 ± 0.1^ab^	9.1 ± 0.4^a^	7.8 ± 0.5^b^	
Ala	3.7 ± 0.1^b^	3.8 ± 0.2^b^	4.4 ± 0.3^ab^	4.0 ± 0.2^b^	4.8 ± 0.4^a^	
Pro	3.6 ± 0.3^b^	4.1 ± 0.1^ab^	3.8 ± 0.3^ab^	3.9 ± 0.3^ab^	4.5 ± 0.3^a^	
Tyr	2.8 ± 0.1^ab^	2.4 ± 0.2^b^	2.5 ± 0.1^ab^	2.9 ± 0.1^a^	2.6 ± 0.3^ab^	
essential amino acids						
Lys	6.2 ± 0.2^a^	6.5 ± 0.1^a^	6.5 ± 0.2^a^	6.6 ± 0.1^a^	6.7 ± 0.3^a^	1.8
Ile	3.1 ± 0.1^b^	3.5 ± 0.2^ab^	3.2 ± 0.2^b^	3.6 ± 0.3^ab^	4.0 ± 0.1^a^	1.5
Leu	6.5 ± 0.2^b^	6.5 ± 0.1^b^	6.6 ± 0.2^b^	6.4 ± 0.1^b^	7.1 ± 0.2^a^	2.1
Phe	5.5 ± 0.1^a^	5.2 ± 0.2^a^	5.4 ± 0.2^a^	5.1 ± 0.5^a^	5.8 ± 0.2^a^	2.1 (Phe + Tyr)
Met	1.3 ± 0.1^b^	1.4 ± 0.0^ab^	1.5 ± 0.1^ab^	1.4 ± 0.2^ab^	1.7 ± 0.1^a^	2.0 (Met + Cys)
Thr	3.2 ± 0.1^a^	3.4 ± 0.1^a^	3.3 ± 0.1^a^	3.2 ± 0.2^a^	3.1 ± 0.3^a^	1.1
Val	3.6 ± 0.2^b^	3.5 ± 0.1^b^	3.6 ± 0.1^b^	3.8 ± 0.2^ab^	4.1 ± 0.1^a^	1.5
His	2.8 ± 0.1^a^	2.5 ± 0.1^ab^	2.4 ± 0.0^ab^	2.6 ± 0.3^ab^	2.3 ± 0.2^b^	1.5
acidic (Asp, Glu)	26.5	29.3	26.7	27.5	28.3	
basic (Lys, Arg, His)	16.9	18.3	17.6	18.3	16.8	
hydrophobic (Ala, Ile, Leu, Met, Phe, Pro, Val)	27.3	28.0	28.5	28.2	32.0	
uncharged polar (Gly, Ser, Thr, Tyr)	14.9	14.3	14.2	14.9	14.1	

a Adapted from Tan et al.^52^

b Data represent the mean ± SD (*n* = 3); Mean values with different letters within the same row are significantly different.

### 3.3 Surface Charge

The net surface charge of the isolates was measured in terms of zeta potential as a function of pH (Table 3). ζ-potential values of proteins show a negative value above the isoelectric point (pI) and a positive value below the pI. Our results agree with those reported by Ladjal-Ettoumi et al.^18^ for isoelectrically precipitated chickpea protein isolates. Protein–water interactions are favored at pH values below or above the pI since the protein in these pH regions has a net charge.^36^ ζ-potential values of chickpea protein isolates range between 16.2 and 29.1 mV at pH 2.0. Azkan, black chickpea, and Erzincan isolates had relatively high surface charges at acidic pH (2.0–3.0). At a neutral pH, ζ-potential varied from −32.5 to −17.7 mV, with the black chickpea isolate showing the highest net charge. Can Karaca et al.^14^ reported the net surface charge of isoelectrically precipitated Kabuli-type chickpea protein isolate to be −22.7 mV at pH 7.0. At pH 10.0, the isolates’ net charge varied between −33.9 and −23.6 mV, where the black chickpea isolate showed the highest surface charge. The pI values of the isolates ranged between pH 4.6 and 4.9 (Table 3).

**Table 3:** Change of ζ-Potential Values of Chickpea Protein Isolates with pH

ζ-potential (mV)					
pH	Hisar	Erzincan	Black chickpea	Azkan	Yasa
2.0	16.2 ± 0.2^e^^a^	18.8 ± 0.3^d^	26.1 ± 0.8^b^	29.1 ± 0.5^a^	20.8 ± 0.7^c^
3.0	21.7 ± 0.2^c^	28.5 ± 0.8^a^	29.0 ± 0.5^a^	25.0 ± 0.2^b^	25.5 ± 0.2^b^
4.0	15.3 ± 0.4^a^	11.1 ± 0.2^c^	12.1 ± 0.3^c^	11.8 ± 0.1^c^	14.0 ± 0.6^b^
5.0	–4.6 ± 0.2^a^	–10.1 ± 0.5^c^	–13.6 ± 0.3^d^	–4.6 ± 0.0^a^	–7.6 ± 0.3^b^
6.0	–13.8 ± 0.2^a^	–17.7 ± 0.7^b^	–26.4 ± 2.0^c^	–14.5 ± 0.1^a^	–15.1 ± 0.3^ab^
7.0	–17.7 ± 0.3^a^	–22.9 ± 0.5^b^	–32.5 ± 1.6^c^	–18.9 ± 0.6^a^	–22.5 ± 0.3^b^
8.0	–19.8 ± 0.7^a^	–26.5 ± 0.5^b^	–32.6 ± 0.5^c^	–25.5 ± 0.1^b^	–24.7 ± 1.0^b^
9.0	–21.6 ± 0.4^a^	–27.0 ± 0.9^b^	–31.6 ± 0.8^c^	–23.5 ± 0.6^a^	–26.3 ± 0.6^b^
10.0	–23.6 ± 0.4^a^	–30.2 ± 0.8^b^	–33.9 ± 2.0^c^	–27.2 ± 0.2^ab^	–28.0 ± 1.5^b^
pI	4.9 ± 0.0^a^	4.7 ± 0.0^c^	4.6 ± 0.0^d^	4.7 ± 0.0^c^	4.8 ± 0.0^b^

a Data represent the mean ± SD (*n* = 3); mean values with different letters within the same row are significantly different.

### 3.4 Thermal Properties

The onset temperature (*T*_o_) of isolates ranged between 36.3 and 115.9 °C, whereas the denaturation temperature (*T*_d_), an indicator of the thermal stability of proteins, ranged from 86.8 to 144.9 °C (Table 4). *T*_d_ of the Erzincan, Azkan, and Yasa isolates were found to be significantly higher than that of the Hisar and black chickpea isolates (*p* < 0.05). There is a wide range of *T*_d_ values reported in the literature for chickpea proteins isolated from different cultivars. For example, in a study by Ghribi et al.^17^ the *T*_d_ of isolates obtained from a Tunisian chickpea cultivar ranged from 127 to 135 °C. In contrast, Kaur & Singh^13^ reported the *T*_d_ of isolates from different Indian chickpea cultivars between 98.5 and 99.8 °C. On the other hand, Withana-Gamage et al.^15^ reported the *T*_d_ ranges from 76.8 to 84.7 °C for proteins isolated from two different chickpea biotypes grown in Canada. The thermal stability of proteins is affected by the balance between polar and nonpolar residues so that proteins with higher amounts of nonpolar residues show higher thermal stability. The *T*_d_ of isolates was found to be positively correlated with the amount of acidic amino acids (*r* = 0.765, *p* < 0.01, Table 5). In general, isolates with relatively higher amounts of acidic amino acids such as Erzincan, Azkan, and Yasa showed higher *T*_d_ values. The denaturation enthalpy (Δ*H*) value measures the amount of heat in the reaction and used as an indication of the extent of protein denaturation during heat treatment. When a protein structure is more regular and not denatured, the Δ*H* increases.^37^ The Δ*H* values of isolates varied between 25.4 and 123.2 J/g. The Hisar isolated showed the highest Δ*H*, followed by the black chickpea isolate. The differences in Δ*H* values of proteins are attributed to the differences in protein structure and composition and the presence of residual salts in the isolates.^13^ Withana-Gamage et al.^15^ reported the Δ*H* value ranges from 2.8 to 3.6 J/g. In another study, the Δ*H* value for chickpea protein isolates was found to be between 2.84 and 5.83 J/g.^13^

**Table 4:** Thermal and Functional Properties of Chickpea Protein Isolates^a^

protein isolate	*T*_o_ (°C)	*T*_d_ (°C)	Δ*H* (J/g)	WHC (g/g)	OHC (g/g)	EC (g/g)	EAI (m^2^/g)	ESI (min)	FC (%)	FS 10 min (%)	FS 30 min (%)	FS 60 min (%)	LGC (g/L)
Hisar	42.6 ± 1.4^b^	88.4 ± 0.4^b^	123.2 ± 3.0^a^	2.1 ± 0.1^b^^b^	4.1 ± 0.3^ab^	467.6 ± 0.8^a^	25.7 ± 0.1^a^	83.8 ± 5.2^b^	68.7 ± 1.2^a^	78.3 ± 2.4^a^	70.7 ± 2.3^a^	66.4 ± 2.1^a^	70 ± 0^c^
Erzincan	115.9 ± 3.8^a^	142.7 ± 2.8^a^	25.4 ± 1.4^d^	2.6 ± 0.1^a^	3.9 ± 0.3^ab^	401.2 ± 0.3^b^	25.4 ± 1.0^a^	146.9 ± 0.8^a^	60.0 ± 4.0^b^	78.1 ± 1.1^a^	72.2 ± 5.9^a^	61.1 ± 2.9^ab^	100 ± 0^b^
Black chickpea	36.3 ± 0.4^c^	86.8 ± 1.2^b^	98.1 ± 1.9^b^	2.7 ± 0.1^b^	3.4 ± 0.2^c^	466.9 ± 2.2^a^	16.8 ± 0.4^b^	45.7 ± 1.9^d^	70.7 ± 1.2^a^	75.3 ± 2.2^a^	67.4 ± 2.3^a^	66.8 ± 1.2^a^	110 ± 0^a^
Azkan	115.5 ± 2.5^a^	144.9 ± 2.9^a^	39.7 ± 0.3^c^	2.1 ± 0.1^b^	4.4 ± 0.1^a^	404.7 ± 0.7^b^	17.4 ± 0.3^b^	63.4 ± 1.8^c^	65.3 ± 2.3^ab^	76.6 ± 3.8^a^	69.5 ± 3.6^a^	59.7 ± 0.2^ab^	100 ± 0^b^
Yasa	114.0 ± 0.8^a^	143.0 ± 3.0^a^	36.6 ± 1.4^c^	2.3 ± 0.0^b^	3.9 ± 0.1^bc^	469.1 ± 1.1^a^	14.5 ± 0.1^c^	69.2 ± 2.5^c^	67.3 ± 3.1^ab^	78.2 ± 2.2^a^	71.2 ± 1.9^a^	55.7 ± 5.1^b^	60 ± 0^d^

a *T*_o_: onset temperature, *T*_d_: denaturation temperature, Δ*H*: denaturation enthalpy, water-holding capacity (WHC), oil-holding capacity (OHC), emulsion capacity (EC), emulsifying activity index (EAI), emulsion stability index (ESI), and least gelation concentration (LGC).

b Data represent a mean ± SD (*n* = 3); mean values with different letters within the same column are significantly different.

**Table 5:** Pearson Correlation Coefficients (*r*) for the Amino-Acid Composition and Physicochemical and Functional Properties of Isolates^a^

	acidic amino acids	basic amino acids	hydrophobic amino acids	uncharged polar amino acids	*T*_d_	WHC	OHC	EC	EAI	ESI	FC	FS 60 min	LGC
acidic amino acids	1												
basic amino acids	0.404	1											
hydrophobic amino acids	0.276	–0.289	1										
polar amino acids	–0.305	0.014	–0.662^c^	1									
*T*_d_	0.765^c^	0.352	0.425	–0.137	1								
WHC	0.170	0.302	–0.023	–0.602^b^	–0.182	1							
OHC	0.065	0.194	–0.192	0.551^b^	0.442	–0.632^b^	1						
EC	–0.540^b^	–0.743^c^	0.322	–0.235	–0.655^c^	–0.029	–0.484	1					
EAI	0.063	0.159	–0.662^c^	0.309	–0.217	0.022	0.295	–0.278	1				
ESI	0.673^c^	0.302	–0.226	–0.069	0.386	0.208	0.244	–0.569^b^	0.731^c^	1			
FC	–0.748^c^	–0.420	0.172	–0.165	–0.628^b^	–0.003	–0.372	0.725^c^	–0.360	–0.745^c^	1		
FS 60 min	–0.683^c^	–0.133	–0.566^b^	0.157	–0.793^c^	0.236	–0.264	0.239	0.397	–0.110	0.366	1	
LGC	–0.010	0.690^c^	–0.506	0.059	–0.087	0.575^b^	–0.237	–0.521^b^	0.035	0.031	–0.154	0.349	1

a *T*_o_: onset temperature, *T*_d_: denaturation temperature, Δ*H*: denaturation enthalpy, water-holding capacity (WHC), oil-holding capacity (OHC), emulsion capacity (EC), emulsifying activity index (EAI), emulsion stability index (ESI), least gelation concentration (LGC).

b Correlation is significant at the 0.05 level.

c Correlation is significant at the 0.01 level.

### 3.5 FTIR Spectrum

**Figure 1:**
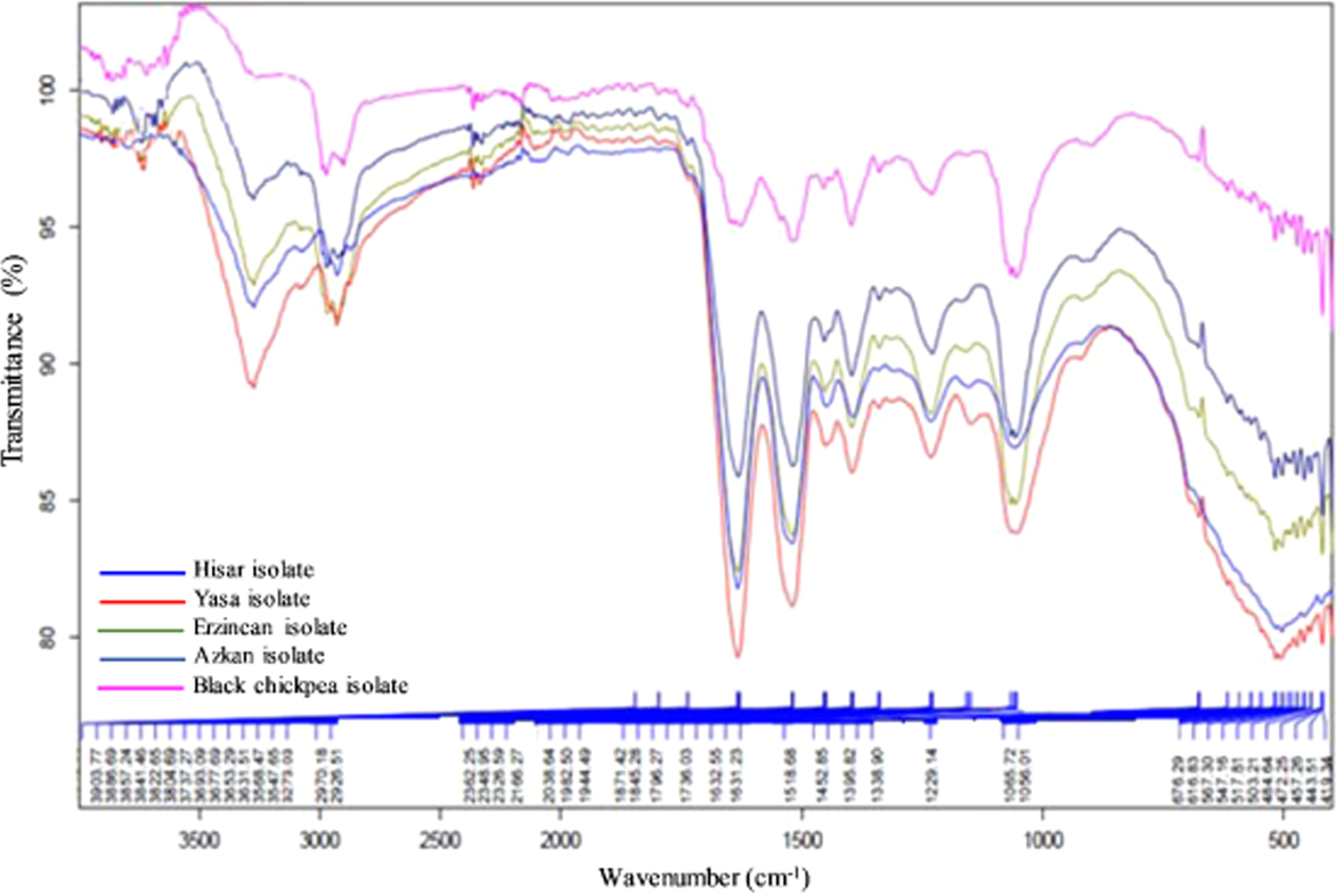
FTIR spectra of chickpea protein isolates in the 400–4000 cm^–1^ band.

The secondary structural features of chickpea protein isolates were determined by FTIR spectroscopy (Figure 1), and the FTIR profile of the studied isolates appeared to be similar. Increased intensity of the bands between 1620 and 1640, 1641 and 1649, and 1650 and 1660 cm^–1^ is associated with an increase in the β-sheet, random winding, and α-helix contents, respectively.^38^ The primary peak was observed in the amide I region between 1627 and 1633 cm^–1^, indicating that the β-sheet structure was dominant in all of the isolates, which is in accordance with the reports of Timilsena and co-workers^38^ and Aryee & Boye.^39^ Withana-Gamage et al.^15^ estimated the secondary structure of Kabuli and Desi chickpea protein isolates to be 33–40% β-sheets, 26–33% α-helices, 14–19% turns, and 16–19% disordered structures. Espinosa-Ramirez and Serna-Saldivar^20^ also reported that the predominant form was β-sheet followed by α-helix for Kabuli-type chickpea protein. The secondary structural components of the proteins are generally observed in the amide I region and the 1610–1700 cm^–1^ band. In this area, approximately 80% of peptide bonds exhibit C=O tensile vibrations, some N–H bending vibrations, and some C–H tensile vibrations.^40^Figure 1 shows another significant peak around wavelength 3273 cm^–1^, which indicates an interaction between protein and water molecules.^15^ The broadband observed at approximately 3302 cm^–1^ corresponds to O–H stretching vibrations, which are primarily caused by water, proteins, and carbohydrates.^41,42^

### 3.6 Solubility

**Figure 2:**
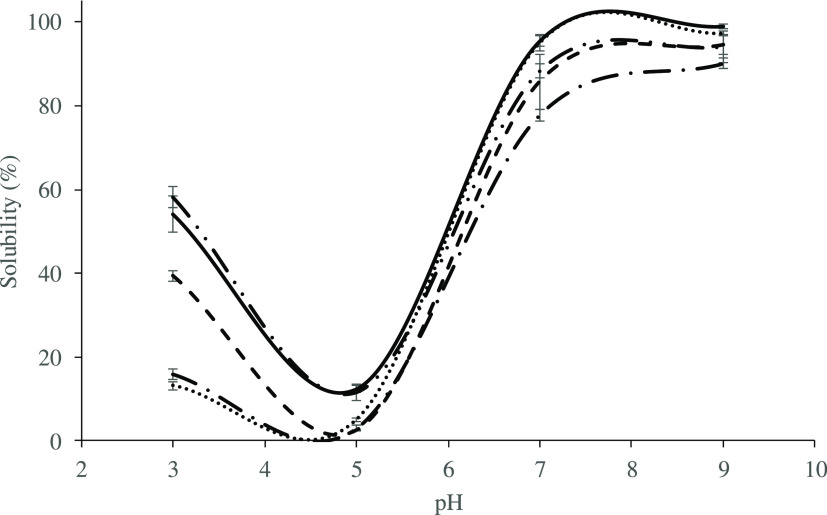
Solubility profile of chickpea protein isolates (−–Hisar, – · Erzincan, - - - Black chickpea, ··· Azkan, – · · Yasa).

Solubility depends on protein composition and structure and is generally accepted as an indicator of protein performance as it affects many other functional properties.^17^Figure 2 presents the change in solubility values of chickpea protein isolates with pH. All isolates were mainly dissolved at pH 9.0 (90.2–99.0%). At pH 7.0, isolate solubility was around ∼88.5%, close to the value (91.2%) previously reported for the isoelectrically precipitated chickpea protein isolate.^14^ All studied isolates showed the lowest solubility at pH 5.0, as this pH value is close to their pI (pH 4.6–4.9). Previous reports by other researchers have also shown the solubility of chickpea protein isolates to be lowest at pH 4–6 and highest between pH 8 and 10.^4,15,17−19^ Solubility of isolates at the acidic pH (3.0) changed within a wide range of 13.2–58.2%. The solubility of Azkan and Erzincan isolates at pH 3.0 (∼14.6%) was significantly lower than that of Black chickpea isolate (39.4%). On the other hand, Hisar and Yasa isolates showed the highest solubility (∼56.2%) at the acidic pH, which could be beneficial in acidic food and beverage applications.

### 3.7 Water-Holding (WHC) and Oil-Holding Capacities (OHC)

WHC is expressed as the amount of water absorbed per gram of protein isolate and indicates the interaction between protein molecules and water.^43^ Bakery products, sauces, and soups are examples of food products in which the WHC of proteins plays an essential role.^13^ OHC is defined as the amount of oil absorbed per gram of protein isolate and is associated with the presence of nonpolar amino acids.^43^ Therefore, OHC plays a vital role in various food applications such as meat products, soups, and bakery products.^13^ The WHC and OHC of chickpea protein isolates are presented in Table 4. The WHC of chickpea protein isolates ranged from 2.1 to 2.7 g/g, with the WHC of Erzincan and black chickpea isolates (2.7 ± 0.2 g/g) found to be significantly higher than that of the other samples (*p* < 0.05). Due to their relatively high WHC, Erzincan and black chickpea isolates can be suggested for bakery products, sauces, and soups. In a recent study, Xing et al.^44^ applied chickpea protein-enriched ingredients in fortification of wheat bread. The authors reported that addition of chickpea protein resulted in improved nutritional profile and firmer and denser structure in bread. Presence of carbohydrates is indicated to affect the hydration properties of proteins.^45,46^ A significant negative correlation was observed between the carbohydrate content and WHC of isolates (*p* < 0.05). The isolates with significantly lower amounts of carbohydrates (Erzincan and black chickpea) were observed to show significantly higher WHC compared to others. On the contrary, isolates with relatively higher amounts of carbohydrates (Hisar, Yasa, and Azkan) showed relatively lower WHC. Moreover, a multiple regression predictive model for WHC identified the amount of uncharged polar amino acids as the significant parameter. The model accounted for 71.2% of the variation found in the data (*p* < 0.05). WHC of isolates was found to be negatively correlated with the amount of uncharged polar amino acids (*r* = – 0.602, *p* < 0.05, Table 5). The WHC of isoelectrically precipitated chickpea protein isolates was reported to be wide-ranging between 2.1 and 7.9 g/g.^13,16,17^

The OHC of chickpea protein isolates ranged between 3.4 and 4.4 g/g. Previous studies have reported the OHC of chickpea protein isolates between 1.3 and 4.1 g/g.^12,13^ OHC of black chickpea isolate (3.4 g/g) was found to be significantly lower than that of Erzincan, Hisar, and Azkan isolates (∼4.2 g/g, *p* < 0.05). Oil absorption properties of plant proteins are affected by protein–lipid–carbohydrate interactions in such a way that presence of carbohydrates may significantly contribute to oil absorption.^47^ In the case of chickpea isolates, a significant positive correlation was observed between the carbohydrate content and OHC (*p* < 0.05). Isolates with significantly higher amounts of carbohydrates (Hisar and Azkan) also showed relatively higher OHC. Furthermore, OHC of isolates was found to be positively correlated with the amount of uncharged polar amino acids (*r* = 0.551, *p* < 0.05) and negatively correlated with WHC (*r* = – 0.632, *p* < 0.05, Table 5).

### 3.8 Emulsifying Properties

Emulsion formation occurs when proteins organize at the oil–water interface, reducing interfacial tension and forming a film around the newly formed oil droplets. Thus, phase separation events such as flocculation and sedimentation are prevented.^4^ The emulsion capacity (EC) of chickpea protein isolates was found to be between 401.2 and 469.1 g/g (Table 4). The EC of Hisar, black chickpea, and Yasa isolates (∼467.9 ± 1.6 g/g) was found to be significantly higher than that of Azkan and Erzincan isolates (∼402.9 ± 1.9 g/g) (*p* < 0.05). The EC of isolates was found to be negatively correlated with the amount of acidic amino acids (*r* = – 0.540, *p* < 0.05), basic amino acids (*r* = – 0.743, *p* < 0.01), and denaturation temperature (*r* = – 0.655, *p* < 0.01, Table 5). A multiple regression predictive model for EC identified the amount of hydrophobic amino acids and *T*_d_ as the significant parameters. The model was able to explain 93.7% of the variation found in the data (*p* < 0.001). The EC of an isoelectrically precipitated chickpea protein isolate was reported to be between 481 and 513 g/g.^14^ Aydemir and Yemenicioglu^16^ observed limited variation in EC of proteins isolated from two different Turkish chickpea cultivars Cevdetbey and Sari, measured by a turbidimetric method. The emulsifying activity index (EAI) of chickpea protein isolates was found to range from 14.5 to 25.7 m^2^/g (Table 4). Hisar and Erzincan isolates showed the highest EAI (∼25.6 ± 0.7 m^2^/g; *p* < 0.05). The EAI of isolates was found to be negatively correlated with the amount of hydrophobic amino acids (*r* = – 0.662, *p* <0.01, Table 5). Isolates with relatively lower amounts of hydrophobic amino acids such as Hisar and Erzincan were observed to show higher EAI values. Boye et al.^4^ reported that EAI (5.7 m^2^/g) of protein concentrates from Canadian desi and Kabuli chickpeas was significantly higher than that of yellow pea, green lentil, and red lentil protein concentrates. It has been indicated that an ideal balance between the hydrophilic and hydrophobic groups is essential for a protein to be an effective emulsifier. In this context, emulsion capacity of isolates was found to be related to the amount of acidic and basic amino acids, whereas emulsifying activity was related to the amount of hydrophobic acids. The emulsion stability index (ESI) of isolates was found to be between 45.7 and 146.9 min (Table 4), with the Erzincan isolate exhibiting the highest emulsion stability (*p* < 0.05). The ESI of isolates was found to be positively correlated with the amount of acidic amino acids (*r* = 0.673, *p* < 0.01, Table 5). Erzincan isolate, which had the highest amount of acidic amino acids among the isolates studied, also showed the highest ESI value. A multiple regression predictive model for ESI identified the amount of acidic and hydrophobic amino acids as the significant parameters. The model accounted for 73.1% of the variation found in the data (*p* < 0.05). Can Karaca et al.^14^ reported the ESI value of isoelectrically precipitated chickpea protein isolate to be 84.9 min. Emulsifying properties of pulse proteins are affected by pulse type, cultivar, protein isolation method, processing conditions, and the technique and conditions used for assessing emulsifying activity and stability.^4,16,19^ A significant positive correlation was observed between the EAI and ESI of isolates (*r* = 0.731, *p* < 0.01, Table 5). Isolates with significantly higher EAI (Hisar and Erzincan) also showed significantly higher ESI values. On the other hand, a negative correlation was found between the ESI and EC of isolates (*r* = – 0.569, *p* < 0.05, Table 5).

### 3.9 Foaming Properties

The ability of proteins to form films while whisking is key to making products such as butter and ice cream. Foaming by proteins is formed by rearranging soluble proteins in the air–water interface and rapid change in protein conformation.^30^ When protein solutions are whipped, proteins form foams due to their surface-active properties.^13^ The foaming capacity (FC) and stability (FS) values of chickpea protein isolates are presented in Table 4. The FC of chickpea protein isolates was between 60.0 and 70.7%. The FC values of the Hisar and black chickpea isolates were found to be significantly higher than that of the Erzincan isolate (*p* < 0.05). A multiple regression predictive model for FC identified the amount of acidic amino acids as the significant parameter. The model was able to explain 79.1% of the variation found in the data (*p* < 0.05). The FC of isolates was found to be negatively correlated with the amount of acidic amino acids (*r* = – 0.748, *p* < 0.01, Table 5). Isolates with relatively lower amount of acidic amino acids including Hisar and black chickpea showed relatively higher FC values. Moreover, a positive correlation was observed between the emulsion capacity and foaming capacity of the isolates (*r* = 0.725, *p* < 0.01, Table 5). Isolates which were able to emulsify higher amounts of oil were able to form foams with higher volume. On the other hand, negative correlations were found between FC and *T*_d_ as well as an ESI. In a study by Kaur & Singh,^13^ the FC of chickpea protein isolates was found to be between 30.4 and 44.3%, and FC was reported to increase with increasing protein concentration. All isolates showed similar FS values after standing for 10 (∼77.3%) and 30 (∼70.2%) min. The FS values of the isolates were found to be between 55.7 and 66.8% at 60 min. The FS values of the Hisar and black chickpea isolates at 60 min were found to be significantly higher than that of the Yasa isolate (*p* < 0.05). Moreover, FS values of isolates at 60 min was found to be negatively correlated with the amount of acidic amino acids (*r* = – 0.683, *p* < 0.01), hydrophobic amino acids (*r* = – 0.566, *p* < 0.05), and *T*_d_ values (*r* = – 0.793, *p* < 0.01, Table 5). Hisar and black chickpea isolates, which had relatively lower amounts of acidic and hydrophobic amino acids, showed relatively higher FS at 60 min.

### 3.10 Gel Formation Capacity

Globular proteins can form gels when subjected to heat treatment. Hydrophobic groups in the protein are essential for forming a gel where they interact and create a three-dimensional network where electrostatic interactions and H bonds are also involved.^48^ Protein isolates with good gelling properties can be used to provide desired textural characteristics in products such as pudding and ice cream. The ability of proteins to form a gel network when heated is affected by the protein concentration, amount of water, ionic strength, time, pH, and temperature.^49^ The least gelation concentration (LGC) is expressed as the lowest protein concentration that can form gels, and proteins with lower LGC values have enhanced gelling capacities.^4^ The LGC of chickpea protein isolates ranged from 60 to 110 g/L (Table 4), whereas the Yasa isolate had the best gelling capacity with an LGC of 60 g/L. The gelling properties of globular proteins are generally affected by thermal characteristics of proteins related to the content of disulfide bonds and hydrophobic amino acids, the heterogeneity of polypeptides, and the hydrophobic interactions among the subunits.^50^ The good gelation properties of the Yasa isolate might be related to its relatively high hydrophobic amino acid content compared to other isolates (Table 2). Nonetheless, the negative correlation between LGC and the amount of hydrophobic amino acids was not found to be significant (*r* = −0.506, *p* > 0.05, Table 5). Besides, no significant correlation was observed between the LGC and *T*_d_ of isolates. A multiple regression predictive model for LGC identified the amount of basic amino acids as the significant parameter. The model was able to explain 67.9% of the variation found in the data (*p* < 0.05). Moreover, positive correlations were observed between LGC and the amount of basic amino acids as well as WHC of isolates. In this sense, Hisar isolate, which had relatively lower amount of basic amino acids and showed lower WHC, was observed to have relatively good gelling capacity. Ghribi et al.^17^ reported the LGC of chickpea protein concentrates on being between 140 and 160 g/L. In other studies, the LGC of isoelectrically precipitated chickpea protein isolates was between 50 and 140 g/L.^4,13,16^ Variations in protein composition, the purity of the obtained protein, drying conditions, and protein extraction conditions are reported to affect gelation.^15^

## 4 Conclusions

Chickpea protein isolates exhibited significant variations in surface charge and thermal and functional properties. According to the FTIR spectra, the β-sheet structure was the primary secondary structure in all isolates. The Hisar and Erzincan isolate exhibited the best emulsifying properties, whereas the Hisar and Black chickpea isolates demonstrated the best foaming properties. No direct relationship was observed between the studied isolates’ amino-acid composition and functional properties except for the Yasa isolate, which had relatively high hydrophobic amino-acid content and showed the highest gelling capacity. The results obtained in this study have thus provided helpful information on the composition, surface charge, and thermal and functional properties of proteins extracted from Anatolian chickpea landraces and their potential applications in various food formulations. Hisar and Yasa isolates can be used in acidic food and beverage applications due to their relatively high solubility at acidic pH. Erzincan and Black chickpea isolates can be suggested for bakery products, sauces, and soups due to their high WHC, whereas Hisar and Azkan isolates with high OHC can be used in meat and bakery products. Hisar isolate also showed good emulsifying properties, foaming capacity, and high oil-holding capacity, which indicated that it could potentially be used in plant-based meat alternatives. On the other hand, considering the fact that raw materials used in this study for protein extraction are specific agricultural products, their relatively limited production quantities can be a challenge toward commercialization. Further research on specific product applications is needed to examine the effects of isolates on the textural properties, appearance, and sensory attributes of end products, which could provide valuable information on their potential utilization as alternatives to commercial proteins.
